# Neutrophil to lymphocyte ratio and platelet to lymphocyte ratio are superior to other inflammation-based prognostic scores in predicting the mortality of patients with gastrointestinal perforation

**DOI:** 10.1186/s40981-017-0118-1

**Published:** 2017-09-02

**Authors:** Yuichiro Shimoyama, Osamu Umegaki, Tomoyuki Agui, Noriko Kadono, Toshiaki Minami

**Affiliations:** 1 Department of Anesthesiology, Osaka Medical College, Intensive Care Unit, Osaka Medical College Hospital, 2-7 Daigaku-machi, Takatsuki, Osaka, 569-8686 Japan; 2 Department of Surgery, Osaka Medical College, Intensive Care Unit, Osaka Medical College Hospital, Takatsuki, Japan; 3 Department of Anesthesiology, Osaka Medical College, Takatsuki, Japan

**Keywords:** Inflammation-based prognostic score, Gastrointestinal perforation, In-hospital mortality

## Abstract

**Background:**

The neutrophil to lymphocyte ratio (NLR) is gaining interest as an independent predictor of survival in patients with various clinical conditions. No study to date has reported an association between inflammation-based prognostic scores, including the Glasgow Prognostic Score (GPS), NLR, platelet to lymphocyte ratio (PLR), Prognostic Nutritional Index (PNI), and Prognostic Index (PI), and mortality in patients with gastrointestinal perforation (GIP). We compared the prognostic value of these measures.

**Findings:**

A total of 32 patients with GIP were retrospectively enrolled. Patients were assessed according to the GPS, NLR, PLR, PI, and PNI. Multivariate analyses were performed to identify variables associated with mortality. Receiver operating characteristic (ROC) analyses were also performed. Overall survival rates (in-hospital mortality) were calculated using the Kaplan–Meier method, and differences in survival rates between groups were compared by the log-rank test. Multivariate analysis of significant variables revealed NLR (HR 1.257, 95% CI 1.035–1.527, *P* = 0.021) and PLR (HR 1.004, 95% CI 1.001–1.007, *P* = 0.016) at the time of admission to the intensive care unit to be independently associated with in-hospital mortality. AUC analysis revealed Sequential Organ Failure Assessment-Glasgow Coma Scale (SOFA-GCS) (0.73) to be superior to NLR (0.57) and PLR (0.58) for predicting mortality, and a high SOFA-GCS score was associated with reduced overall survival (*P* < 0.05).

**Conclusions:**

NLR and PLR were superior to other inflammation-based prognostic scores in predicting the mortality of patients with GIP.

## Findings

### Introduction

The neutrophil to lymphocyte ratio (NLR) has gained interest as an independent predictor of survival in patients with various clinical conditions, ranging from oncological to cardiovascular diseases. NLR has also been reported to predict bacteremia better than other infection markers [1], and an NLR > 7 was reportedly an independent marker of mortality in patients with bacteremia [2].

Gastrointestinal perforation (GIP) is a life-threatening disease with a high mortality rate; GIP often leads to shock and usually requires active rescue in the intensive care unit (ICU) and emergency laparotomy [3]. No previous study has reported an association between inflammation-based prognostic scores and outcomes in patients with GIP.

We hypothesized that NLR measured at the time of admission to the ICU may better predict in-hospital mortality in patients with GIP, as compared with other inflammation-based prognostic scores. To test this hypothesis, we compared the prognostic value of various inflammation-based prognostic scores in patients with GIP.

### Methods

We conducted a single-center retrospective study in a 16-bed ICU. The study protocol was approved by the Ethics Committee of Osaka Medical College (Osaka, Japan). A total of 40 patients diagnosed with GIP, who underwent surgery and were treated in the ICU of Osaka Medical College Hospital between January 2014 and June 2016, were retrospectively enrolled. Of these, 32 patients were evaluated, excluding those who were aged 18 years or younger; who were pregnant; who had immunosuppressive disease (e.g., HIV), or were undergoing immunosuppressive therapy (e.g., chemotherapy, chronic use of steroids, autoimmune disease treatment) within 1 month of the study; and who had cardiac arrest at the time of ICU admission. Individual patient consent was not obtained since all data used in this study were acquired retrospectively from the laboratory information system without any additional blood sampling or laboratory analysis. The main outcome measure was in-hospital mortality. The following demographic and clinical data were collected: age, sex, comorbidities (cancer, coronary artery disease, diabetes mellitus, hypertension, and renal disease), Sequential Organ Failure Assessment-Glasgow Coma Scale (SOFA-GCS) score at ICU day 1, length of hospital stay, and in-hospital mortality. Since our study population included intubated patients under sedation with propofol and/or dexmedetomidine at ICU admission, we excluded the GCS item from the SOFA score. Blood samples were obtained upon ICU admission for measurements of CRP, albumin, white blood cell count, neutrophil count, lymphocyte count, and platelet count. The GPS, NLR, PLR, PI, and PNI were obtained as shown in Table 1.

**Table 1:** Inflammation-based prognostic scores

Scoring systems	Score
Glasgow Prognostic Score	
CRP (≤ 10 mg l^−1^) and albumin (≥ 35 g l^−1^)	0
CRP (≤ 10 mg l^−1^) and albumin (< 35 g l^−1^)	1
CRP (> 10 mg l^−1^) and albumin (≥ 35 g l^−1^)	1
CRP **(>** 10 mg l^−1^) and albumin (< 35 g l^−1^)	2
Neutrophil to lymphocyte ratio	
Neutrophil count: lymphocyte count	
Plt to lymphocyte ratio	
Plt count: lymphocyte count	
Prognostic Index	
CRP (≤ 10 mg l^−1^) and white blood cell count (≤ 11 × 10^9^ l^−1^)	0
CRP (≤ 10 mg l^−1^) and white blood cell count (> 11 × 10^9^ l^−1^)	1
CRP (> 10 mg l^−1^) and white blood cell count (≤ 11 × 10^9^ l^−1^)	1
CRP (>10 mg l^−1^) and white blood cell count (>11 × 10^9^ l^−1^)	2
Prognostic Nutritional Index	
Albumin (g l^−1^) + 5 × total lymphocyte count 10^9^ l^−1^	

*CRP* C-reactive protein, *Plt* platelet

Descriptive analysis was performed for all variables. Continuous variables were expressed as median (interquartile range), and categorical variables as counts (percentage). Patient characteristics were compared between survivors and non-survivors using Fischer’s exact test. Univariate analysis and multivariate analysis (Cox proportional hazards model) were used to examine associations between patient characteristics and prognostic factors. Analyses using Cox proportional hazards models were performed by forward selection of variables which were found to be significant by univariate analysis and inflammation-based prognostic scores. Receiver operating characteristics (ROC) curves were generated for variables which were significant in the multivariate analysis, and areas under the curve (AUCs), cutoff values, sensitivities, specificities, and predictive values were calculated. Using these cutoff values, overall survival rates (in-hospital mortality) were calculated with the Kaplan–Meier method, and differences in survival rates between groups were compared by the log-rank test. A *P* value < 0.05 was considered statistically significant. All statistical analyses were performed using the BellCurve for Excel software package v.2.0 (Social Survey Research Information Co., Ltd., Tokyo, Japan).

### Results

Baseline characteristics of the patients are shown in Tables 2 and 3. Twenty-four (75%) patients were survivors (perforation in the colon, 16; small intestine, 6; stomach, 1; appendix, 1), and 8 (25%) were non-survivors (colon, 4; small intestine, 4). The median age was 74 (range, 65.5–79) years for survivors and 66.5 (range, 64.5–73) years for non-survivors. Among survivors, 10 (41.7%) patients were males and 14 (58.3%) were females, and among non-survivors, 4 (50%) were males and 4 (50%) were females.

**Table 2:** Patient demographics

	All patients		Univariate analysis
	Survivors	Non-survivors	*P* value
Variables	(*n* = 24)	(*n* = 8)	
Age, year	74 (65.5–79)	66.5 (64.5–73)	0.58
Female	14 (58.3)	4 (50)	0.62
Male	10 (41.7)	4 (50)	
Cancer	16 (67)	6 (75)	0.69
CAD	1	0	
Diabetes	4	0	
Hypertension	8	0	
Renal disease	1 (4)	3 (38)	0.039
Observation period	36 (24–46.5)	20.5 (13.8–25.8)	0.0069
Albumin (g l^−1^)	19 (14.8–24)	23 (13.8–28.3)	0.406
CRP (mg l^−1^)	11.5 (8.3–18)	9.1 (5.4–15)	0.809
WBC (× 10^9^ l^−1^)	6.3 (3.6–8.4)	4.8 (3.8–6.8)	0.552
Neutrophil count (× 10^9^ l^−1^)	5.3 (2.9–7.2)	3.8 (3.2–4.9)	0.454
Lymphocyte count (× 10^9^ l^−1^)	0.49 (0.36–0.78)	0.31 (0.28–0.37)	0.064
Plt count (× 10^4^ mm^−3^)	17.3 (15.2–23.2)	21.1 (8.0–29)	0.484

*CAD* coronary artery disease, *CRP* C-reactive protein, *WBC* white blood cell, *Plt* platelet

**Table 3:** Inflammation-based prognostic scores

	All patients		Univariate analysis
	Survivors	Non-survivors	*P* value
Variables	(*n* = 24)	(*n* = 8)	
SOFA-GCS score at ICU admission	3 (2–4.25)	6.5 (4.8–8.3)	0.0087
GPS (0/1/2)	(0/8/16)	(0/5/3)	0.161
NLR	8.7 (6.4–14.9)	13.7 (7.7–15.6)	0.432
PLR	390.2 (279.8–688.5)	596.2 (274.8–783.8)	0.057
PI (0/1/2)	(8/12/4)	(4/4/0)	0.262
PNI	20.6 (17.4–25.8)	24.3 (15.4–29.8)	0.611

*SOFA* Sequential Organ Failure Assessment, *GCS* Glasgow Coma Scale, *ICU* intensive care unit, *GPS* Glasgow Prognostic Score, *NLR* neutrophil to lymphocyte ratio, *PLR* platelet to lymphocyte ratio, *PI* Prognostic Index, *PNI* Prognostic Nutritional Index

**Fig. 1:**
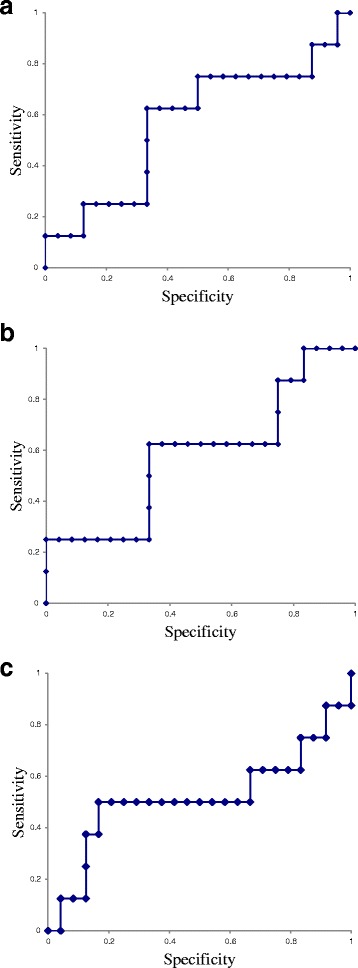
Receiver operating characteristic curves of inflammation-based prognostic scores for predicting mortality. **a** NLR. **b** PLR. **c** SOFA-GCS

**Fig. 2:**
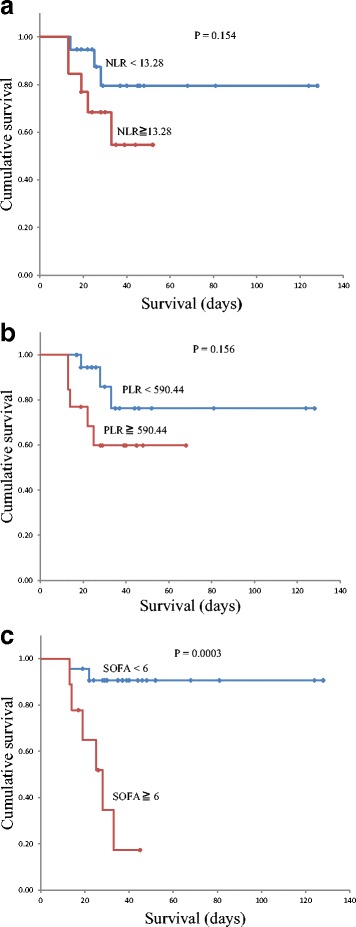
Kaplan-Meier survival curves for inflammation-based prognostic scores. **a** NLR. **b** PLR. **c** SOFA-GCS

Multivariate Cox proportional hazards models revealed NLR (HR 1.257, 95% CI 1.035–1.527, *P* = 0.021) and PLR (HR 1.004, 95% CI 1.001–1.007, *P* = 0.016) to be independently associated with in-hospital mortality (Table 4). Cutoff values for mortality obtained from ROC analysis (Fig. 1) were 13.28 (sensitivity, 62.5%; specificity, 66.7%; area under the curve (AUC), 0.57; 95% CI, 0.31–0.83; *P* = 0.607) for NLR and 590.44 (sensitivity, 62.5%; specificity, 66.7%; AUC, 0.58; 95% CI, 0.33–0.84; *P* = 0.521) for PLR (Tables 5 and 6). AUC analyses revealed SOFA-GCS (0.73) to be superior to NLR (0.57) and PLR (0.58) for predicting mortality (Table 6). A high SOFA-GCS score was associated with reduced overall survival (*P* < 0.05) (Fig. 2).

**Table 4:** Predictors of mortality by multivariate analysis

Predictors	Hazard ratio	95% CI	*P* value
SOFA-GCS score	1.709	1.108–2.637	0.015
NLR	1.257	1.035–1.527	0.021
PLR	1.004	1.001–1.007	0.016
Renal disease	1.238	0.13–11.821	0.853

*SOFA* Sequential Organ Failure Assessment, *GCS* Glasgow Coma Scale, *NLR* neutrophil to lymphocyte ratio, *PLR* platelet to lymphocyte ratio

**Table 5:** Performance parameters for predictors of mortality

Predictors	Cutoff value	Sensitivity (%)	Specificity (%)	PPV (%)	NPV (%)
SOFA-GCS	6	75	87.5	66.7	91.3
NLR	13.28	62.5	66.7	38.5	84.2
PLR	590.44	62.5	66.7	38.5	84.2

*PPV* positive predictive value, *NPV* negative predictive value, *SOFA* Sequential Organ Failure Assessment, *GCS* Glasgow Coma Scale, *NLR* neutrophil to lymphocyte ratio, *PLR* platelet to lymphocyte ratio

**Table 6:** Comparison of AUC between predictors

Predictors	AUC	95% CI	*P* value
SOFA-GCS	0.73	0.44–1.02	0.112
NLR	0.57	0.31–0.83	0.607
PLR	0.58	0.33–0.84	0.521

*AUC* area under the curve, *SOFA* Sequential Organ Failure Assessment, *GCS* Glasgow Coma Scale, *NLR* neutrophil to lymphocyte ratio, *PLR* platelet to lymphocyte ratio

### Discussion

NLR and PLR were found to be superior to other inflammation-based prognostic scores in predicting the mortality of patients with GIP. NLR and PLR are based primarily on the physiological link between neutrophilia and lymphopenia with systemic inflammation. Jilma et al. [4] studied changes in white blood cell types after inflammation and reported a 300% increase in circulating neutrophils, 96% decrease in monocytes, and 85% decrease in lymphocytes 4 to 6 h after inflammation. Below, we discuss the literature surrounding NLR and the prognostic capabilities of NLR for GIP.

Growing evidence suggests the usefulness of NLR in the prediction of survival in various contexts, such as lung cancer, colorectal cancer, orthotopic liver transplantation for primary hepatocellular carcinoma, postoperative coronary artery bypass grafting, chronic heart failure, pulmonary emboli, and acute pancreatitis [1, 5, 6]. Moreover, NLR was a more sensitive parameter than increased white blood cell count in patients with suspected appendicitis [7]. These data suggest the importance of NLR in multiple patient populations.

In the present study, NLR and PLR had a positive predictive value of 38.5% and a negative predictive value of 84.2%, suggesting that NLR and PLR may be more useful for ruling out mortality, rather than predicting it. NLR and PLR can be obtained easily, cheaply, and rapidly and can provide relevant information for necessary interventions within the first few hours of hospital admission. As discussed earlier, studies have shown that NLR predicts bacteremia better than other infection markers [1] and an NLR > 7 was reportedly an independent predictor of mortality in patients with bacteremia [2]. In another study, the initial NLR measured at ED admission was independently associated with 28-day mortality in patients with severe sepsis and septic shock [8]. However, these previous studies did not assess associations between inflammation-based prognostic scores, including the GPS, NLR, PLR, PNI, and PI, and mortality in patients with GIP. Our results are informative in this respect.

This study has a potential limitation. Given the retrospective, single-center design of the study and small cohort, multivariate analysis may be difficult to apply. A large-scale prospective validation study will be needed to confirm our results.

### Conclusion

NLR and PLR were superior to other inflammation-based prognostic scores in predicting the mortality of patients with GIP.

## Abbreviations

AUC: Area under the curve
CRP: C-reactive protein
GCS: Glasgow Coma Scale
GIP: Gastrointestinal perforation
GPS: Glasgow Prognostic Score
ICU: Intensive care unit
NLR: Neutrophil to lymphocyte ratio
PI: Prognostic Index
PLR: Platelet to lymphocyte ratio
PNI: Prognostic Nutritional Index
ROC: Receiver operating characteristics
SOFA: Sequential Organ Failure Assessment
